# Non -fatal overdose among people who inject drugs in Tehran, Iran

**DOI:** 10.1186/s13011-020-00323-0

**Published:** 2020-10-14

**Authors:** Mehdi Noroozi, Peter Higgs, Azadeh Bayani, Bahram Armoon, Ali Nazeri Astaneh, Ladan Fattah Moghaddam, Mohammad Askari

**Affiliations:** 1 Social Determinants of Health Research Center, University of Social Welfare and Rehabilitation Sciences, Tehran, Iran; 2 Department of Public Health, School of Psychology and Public Health, La Trobe University, Melbourne, Australia; 3 Student Research Committee, School of Allied Medical Sciences, Shahid Beheshti University of Medical Sciences, Tehran, Iran; 4 Social Determinants of Health Research Center, Saveh University of Medical Sciences, Saveh, Iran; 5 Department of Psychiatry, University of Social Welfare and Rehabilitation Science, Tehran, Iran; 6 Department of Nursing, Faculty of Nursing and Midwifery, Tehran Medical Sciences, Islamic Azad University, Tehran, Iran; 7 Pasteur institute of Iran, Tehran, Iran

**Keywords:** Non-fatal, Overdose, People who inject drugs, Methamphetamine, Alcohol use

## Abstract

**Background:**

With increasing frequencies of non-fatal overdose in people who inject drugs (PWID), it is essential to improve our knowledge about associated risk factors for overdose to inform overdose prevention and assistance programs. The aim of present study was to determine the prevalence of non-fatal overdose and the associated risk factors among PWID in Tehran, Iran.

**Methods:**

Snowball sampling was used to collect data from 465 participants in Tehran using a cross-sectional survey. Consenting participants who reported drug injecting in the past month and were able to speak and comprehend Farsi enough to respond to survey questions were interviewed. The endpoint of interest was non-fatal overdose in the previous 6 months, or answering “Yes” to the question: “In the last six months, have you ever overdosed by accident? (at least once)”. We used STATA v. 14 for this analysis. Statistical significance was defined as *p* < 0.05 for all analyses.

**Results:**

Of 465 PWIDs who participated in this study, all were male, and about half had less than a high school education. The prevalence of self-reported non-fatal overdose in the past 6 months was 38% (CI95%: 34, 43%). Our findings indicate that characteristics and behaviors that were associated with an increased risk of experiencing an overdose in the past 6 months were drug use initiation under 22 years (AOR =2.2, *P* < 0.05), using methamphetamine (AOR =2.8, *P <* 0.05), and using multiple drugs at the same time (AOR =2.1, *P <* 0.05). Also, more recent initiates to injecting (< 2 years) had an increased risk of experiencing an overdose in the past 6 months. The odds of experiencing a non-fatal overdose among PWIDs who regularly attended NSP were 0.6 times less than for those who did not attend regularly (OR = 0.6,95% CI: 0.2–0.9).

**Conclusion:**

Methamphetamine and alcohol use were the most significant association for non-fatal overdose among PWIDs. Our results indicate that intervention and prevention initiatives seeking to reduce overdoses among PWIDs should not only be focused on the primary drug used but also the use of alcohol and poly-drug use.

## Background

Global estimates suggest there are 16 million people who inject drugs (PWIDs) [1] and drug injection is one of the most important public health issues currently facing Iran [2]. According to local studies, it is estimated that there are between 170,000 and 230,000 PWIDs with around 15% of them are infected with HIV [3]. Overdose accounts for almost one-third of drug-associated deaths among opiate using PWIDs [4, 5] and is increasing across different contexts. Studies suggest more than two thirds of drug users have experienced at least one non-fatal overdose in their life [6] with more than two million drug-related emergency department visits having occurred in 2004 [7].

Considerable morbidity has been associated with non-fatal overdose including physical injuries, aspiration-related lung injury and infections, seizures, and peripheral neuropathy [8]. Previous experience of a non-fatal overdose is a significant risk factor for future overdose (both fatal and non-fatal) and is related with several health risks such as cognitive impairment and muscular dysfunction also high healthcare costs [9, 10]. Studies has also recorded important overlap between the mutual relationship [10–12] of fatal and non-fatal overdose [13]. Other correlates of non-fatal overdose include “polysubstance use” for example, taking multiple kinds of substances that can act together to increase the risk of overdose, such as the simultaneous use of opioids and alcohol, or opioids and benzodiazepines, as well as other factors such as homelessness, injecting in public places such as streets or abandoned houses, and police encounters [11–15]. Few previous studies in Iran investigated the prevalence of non-fatal overdose and their associated risk factors [16, 17]. With non-fatal overdose increasing among PWIDs, it is essential to improve our knowledge about this problem and their associated risk factors to inform overdose prevention and assistance programs. The primary aims of our study was to determine the prevalence of non-fatal overdose and any associated risk factors among PWIDs in Tehran, Iran.

## Methods

The study population and data collection procedures have been described in detail elsewhere [18] but briefly we outline the process below.

### Study design

A cross-sectional study was conducted among current PWIDs to assess prevalence and risk factors for recent overdose in Tehran, in 2016.

### Dependent variable

Self-reported non-fatal overdose in the last 6 months.

### Study sampling

Our final sample size was a total of 485 and we excluded 20 individuals because of not responding to the questions or dissatisfaction with participating in the study.

465 PWIDs were recruited using snowball sampling and convenience sampling. Eligible participants were then given the opportunity to invite their peers to also participate in the study – all participants were reimbursed 15,000 Tomans (Iranian currency) for their involvement in the study.

### Inclusion and exclusion criteria

To be eligible for the study, participants were required to be over 18 years old and to have injected illicit drugs at least once in the past month. Additional eligibility criteria were ability to speak and comprehend Farsi enough to respond to survey questions, and to provide informed consent.

### Study instruments and procedure

Face-to-face interviews were conducted by trained interviewers using a structured questionnaire. Interviews included socio-demographic information (i.e., age, educational attainment, marital status, income and employment status), drug use history (i.e., age of initiation, past 6 months use of specific drugs including heroin, methamphetamine, prescription drugs, cannabis), history of prison, needle syringe program (NSP) exposure, use of poly drugs and alcohol use. All behavioral questions referred to the 6-months prior to completing the interview. Alpha test of the internal consistency of the questionnaire among demonstrated Cronbach’s a values between 0.88 and 0.90. No identifying information was collected from questionnaire respondents.

### Ethics approval and consent to participate

Approval to conduct the study was granted by the Ethics Committee of University of Social Welfare and Rehabilitation Sciences. Informed written consent was received from all participants. The Ethical code was IR.USWR.REC.1398.086.

### Outcome definition

Overdose has been defined as an action with the following characteristics: the loss of consciousness, presenting blue skin color, collapsing, inability to wake up, encountering convulsions, experiencing difficulties with breathing, myocardial infarction, or even death occurred during drug use. This definition was in line with the studies conducted in Adelaide, Australia [19], and San Francisco, California [20]. We created a list of above mentioned characteristics and in cases that participants indicated any of these characteristics we considered them as PWIDs who experienced overdose. We asked study participants to report on any overdose experience over the past 6 months using this definition.

### Statistical analysis

Descriptive statistics were used to characterize the demographics, drug use histories and overdose histories of the overall study population. Firstly, we considered the bivariable relationships between all independent variables and the prevalence of non-fatal overdose using Pearson’s Chi-square test. After checking for collinearity, variables with *p*-value < 0.2 were included in the multiple logistic regression model. Then, variables were eliminated from the multivariable models using stepwise selection. The final model included only variables with *p* < 0.05. We reported the adjusted odds ratio (aOR) point estimate and 95% confidence interval (95% CI) as the effect measure. We used STATA v. 14 for this analysis. Statistical significance was defined as *p* < 0.05 for all analyses.

## Results

Of 465 PWIDs who participated in this study, all were male, and about half had less than a high school education.

The prevalence of non-fatal overdose in the past 6 months was 38% (95% CI: 34, 43%). The main socio-demographic characteristics of PWIDs who reported overdose in comparison with PWIDs who did not reported any overdose are shown in Table 1.

**Table 1:** Characteristics of people who inject drugs and overdose history, Tehran, Iran 2016

Characteristics	Self-reported overdose past six months		*P*-value
	Yes (*n* = 180)	No (*n* = 285)	
	N (%)	N (%)	
Age (year)			
< 30	103 (57)	171 (60)	0.04
30–39	54 (30)	77 (27)	
40+	23 (13)	37 (13)	
Age (Mean + SD)	27.4 ± 7.8	33.2 ± 7.2	0.04
Education			
> High school	108 (60)	145 (51)	0.05
< High school	72 (40)	140 (49)	
Marital status			
Single	94 (52)	134 (47)	0.27
Married	86 (48)	151 (53)	
Employment Status			
Unemployed	99 (55)	134 (47)	0. 09
Employed	81 (45)	151 (53)	
Income (USD)			
> 150	68 (38)	128 (45)	0.12
< 150	112 (72)	157 (55)	
Methamphetamine use			
Yes	86 (48)	95 (33)	0.002
No	94 (52)	190 (67)	
Alcohol use			
Yes	80 (45)	100 (35)	0.04
No	100 (55)	185 (65)	
Age of drug initiation (year)			
< 22	101 (56)	128 (45)	0.01
22+	79 (44)	157 (55)	
Age of onset to injection			
< 22	117 (65)	100 (35)	0.001
22+	63 (35)	185 (65)	
Duration of inject drug			
≤ 2	94 (52)	95 (33)	0.001
> 2	86 (48)	190 (67)	
Poly drug			0.08
Yes	81 (45)	105 (37)	
No	99 (55)	180 (63)	
History of prison			
Yes	45 (25)	57 (20)	0.2
No	135 (75)	228 (80)	
Needle and syringe Program exposure (counseling service)			
Regular	71 (40)	180 (63)	0.001
Irregular	109 (60)	105 (37)	

### Bivariate analyses

In bivariate analyses, there were a number of statistically significant differences in socio- economic characteristics (age) and drug use characteristics between those who had and had not witnessed an overdose.

Participants who reported a history of overdose compared to those who did not were significantly more likely to have started their drug use before the age of 22. They also reported an injecting drug use career of less 2 years. They were using the NSP regularly, were alcohol and methamphetamine users.

### Multiple logistic regression analyses

In the final multiple logistic regression model (Table 2) the characteristics and behaviors that were associated with an increased risk of experiencing an overdose in the past 6 months are presented. There were no significant associations between non-fatal overdose and socio- economic characteristics (age, education and income).

**Table 2:** Multiple logistic regression and Adjust Odds Ratio (AOR) of factors associated with non-fatal overdose among PWID (last 6 months)

Characteristics	Over dose		*P*-Value
	AOR	(% CI95)	
Age (year)			
< 30	1	..........	–
30–39	1.3	(0.1–5.3)	0.3
40+	1.4	(0.2–4.8)	
Education			
> High school	1	..........	–
< High school	1.3	(0.8–2.31)	0.4
Income (USD)			
> 150	1	..........	–
< 150	1.65	(0.93–2.99)	0.3
Age of drug initiation (year)			
< 20	2.2	(1.8–5.7)	0.01
20+	1	–	–
Duration of inject drug			
< 2	2.7	(1.6–4.61)	0.02
> 2	1	..........	–
Methamphetamine use			
Yes	2.8	(1.8–7.4)	0.02
No	1	..........	–
Poly Drug			
Yes	2.1	(1.4–5.3)	
No	1	..........	0.01
Alcohol use			
Yes	2.8	(1.2–4.3)	0.01
No	1	..........	–
Needle and syringe Program exposure			
Regular use of NSP	0.6	(0.2–0.9)	0.01
Irregular use of NSP	1	–	–

Age under 20 years (with those who are younger being at higher risk) was significantly associated with overdose (AOR =2.2, 95% CI: 1.8–5.7, *P* < 0.01). Results showed that starting injecting within the last 2 years was associated with an increased risk of experiencing an overdose in the past 6 months. Recent recruits to injecting were 2.7 times more likely to have had an overdose (AOR 2.7; 95% CI 1.6–4.61, *P* < 0.02).

Methamphetamine use was also positively associated with non-fatal overdose among PWID in this study. Individuals using methamphetamine (AOR 2.8; 95% CI 1.8–7.4, *P <* 0.02) were 2.8 times more likely to have had an overdose in the previous 6 months.

Our results indicates a significant correlation between poly drug use and overdose. Those PWIDs who reported using multiple drugs at the same time were 2.1 times more likely to have had an overdose (AOR 2.1; 95% CI 1.4–5.3, *P* < 0.01).

Our results demonstrated that alcohol use was positively associated with overdose among PWIDs (AOR =2.8, 95% CI: 1.2–4.3, *P* < 0.01).

Finally, our data found that regular NSP use was negatively associated with overdose. Where those attending NSP were 0.6 times less likely to report overdosing than other participants (AOR =0.6, 95% CI: 0.2–0.9, *P <* 0.01).

## Discussion

This study assessed the socio-demographic and behavioral risk factors associated with overdose among PWIDs in Tehran. After adjustment, our findings indicate that PWIDs who started using any illicit drug before the age of 20 were more likely to report experiencing an overdose in the 6 months before their interview. The age that individuals start using opioids is also important to know about and understand because evidence suggests those who begin using at an earlier age are more susceptible to drug dependence and other associated social and/or health problems [21–26]. Consistent with previous studies [27, 28] our analysis suggests that the participants who started injecting less than 2 years before interview (new injectors), were more likely to report a non-fatal overdose. One possible explanation for this is that those with less injecting experience and lower tolerance are more susceptible. However, this explanation is not universally supported, with other studies noting older injectors are more likely to experience recent overdose suggesting that the risk of overdose increases alongside the length of the drug-using career [13, 29].

Our research establishes that PWIDs who are also using other drugs (poly drug use) in the 6 months before interview was more susceptible to experiencing non-fatal overdose. This is not surprising with a number of previous studies showing poly-drug use is strongly associated with both fatal and non-fatal overdose [13, 20, 29]. These data also highlight the broader challenges faced by polydrug users including living on the streets and exposure to structural issues including violence [30].

Our findings that PWIDs who use alcohol and/or methamphetamine were more likely to report a recent overdose are supported by other empirical data [11, 31]. Previous studies have reported higher prevalence of overdose among injection drug users who also report alcohol consumption [31, 32] demonstrating the pharmacological or behavioral interactions between alcohol and injected substances [33].

It is essential to emphasise the significant effect of alcohol in this regard. Alcohol use was commonly reported by participants in our study and prior investigations also indicate an association between greater rates of overdose and alcohol consumption [31, 32].

These data indicate the importance of addressing both drug use and the consumption of alcohol in any harm reduction prevention programs developed to work with PWID in Iran.

The most significant correlation was observed between the use of methamphetamine (injection & non-injection) and overdose. The increasing prevalence of methamphetamine use in Iran may help to explain this finding [34, 35].

Based on our findings, PWIDs attending NSP were at lower risk of overdose. Our finding is in line with other studies [36, 37] showing that the provision of other services from needle and syringe programs, including overdose education and providing naloxone can prevent overdose, [37]. Also, other services include, providing counseling and testing PWIDs about substance abuse treatment, HIV, hepatitis C virus (HCV), HBV counseling and testing [38–43], and naloxone for overdose educating PWIDs [44]. Additionally, integrating the health care systems with the services for PWIDs has positive influences both for PWIDs and the society [45, 46]. It is important to consider that NSP can have significant influences on PWIDs programs. NSP as secondary prevention programs, selling non-prescript syringes in pharmacies, safe dumping of used equipment in injections, and overdose prevention programs among the community, can decrease the risk of injection drug intake and reduce social and medical costs [36, 47, 48].

### Limitation of study

There are some limitations to our study. As this was a cross-sectional study, no causal inference between overdose and risk factors can be made. Our data are based on participant’s self-report and therefore may be subject to misclassification, recall and/or social desirability bias. Furthermore, there was no specific definition for overdose so participant understanding of overdose may not be the same. Limitation was that the obtained data may not be generalized to other groups of PWIDs, is not a random study sample. Besides, the required information was collected on a self-report basis; thus, socially-desired responses are possible, leading to underestimating the non-fatal overdose rate., Another biasing was that, we probed data concerning overdose and disregarded signifying the overdose symptoms in the interviews, e.g., experiencing convulsions and/or the lack of consciousness; due to the use of too strong drugs and the lack of recalling the incidence, PWIDs might have underreported overdose. Eventually, characteristic in the present research was mortality, as we only interviewed the overdose survivors.

## Conclusion

Methamphetamine and alcohol use were the most significant associations for non-fatal overdose among PWIDs in this study. Our findings indicate that any intervention and prevention measures seeking to reduce overdose among PWIDs must address the primary drug being used but also pay attention to the use of alcohol and any other drugs. PWID are more likely than health care workers to be in a position to manage and respond to overdose therefore they must be targeted with overdose prevention education, and trained in the use of naloxone [49]. Harm reduction programs that focus on working with vulnerable and hard-to-reach injection drug users must include the provision of training regarding overdose.

## Data Availability

The datasets used and/or analyzed during the current study are available from the corresponding author on reasonable request.

## Abbreviations

aOR: Adjusted odds ratio
CI: Confidence interval
NSP: Needle and syringe program
OD: Overdose
PWIDs: People who inject drugs
